# Training a medical workforce to meet the needs of diverse minority communities

**DOI:** 10.1186/s12909-017-0858-7

**Published:** 2017-01-21

**Authors:** Faafetai Sopoaga, Tony Zaharic, Jesse Kokaua, Sahra Covello

**Affiliations:** 1 Department Preventive and Social Medicine, Dunedin School of Medicine, University of Otago, P.O. Box 913, , Post Code 9054 Dunedin, New Zealand; 2 Department of Biochemistry, University of Otago, P.O. Box 56, , Post Code 9054, Dunedin, New Zealand; 3 Pacific Islands Research & Student Support Unit, Division of Health Sciences, University of Otago, P.O. Box 56, , Post Code 9054 Dunedin, New Zealand

**Keywords:** Culture, Cultural competency, Immersion programme, Diversity, Medical students, Pacific health

## Abstract

**Background:**

The growing demand for a competent health workforce to meet the needs of increasingly diverse societies has been widely acknowledged. One medical school in New Zealand explored the integration of the commonly used patient-centred model approach, with an intersectional framework in the development of a cultural competency training programme. In the Pacific Immersion Programme, medical students in their fourth year of training are given the opportunity to learn about different factors that influence the health and health care of a minority community through immersion in that community. The programme objectives include enabling students to learn through experience living within the local community context, and supporting them to re-evaluate their own personal beliefs, assumptions and/or prior prejudices. This study evaluates the usefulness of this programme in the training of medical students to work in diverse communities.

**Methods:**

Two analytical approaches were used for evaluation. Deductive and inductive analyses were conducted on 235 reflective essays completed by three cohorts of students from 2011 to 2013 to ascertain the value of the programme for student learning. In addition, one cohort was invited to complete a pre and post-programme questionnaire.

**Results:**

Overall, the students found the programme to be a valued learning environment. They found living within a Pacific family environment to be an eye opening experience. It increased students comfort level in cross cultural engagement and emphasised the importance of patient’s perspectives in health care provision. Students’ self-reported knowledge about Pacific cultural values, protocols, traditional beliefs and the main health challenges increased significantly after the programme. They appreciated learning directly from community members, and through observations about how culture, beliefs and the socio-economic environment influence peoples’ health and wellbeing.

**Conclusions:**

Medical schools are required to train a competent health workforce to meet the needs of diverse communities. The Pacific Immersion Programme provides a unique learning environment which can improve the training of doctors to work in diverse communities. The key to its success is enabling students to be engaged learners from “the inside” rather than an “outsider looking in”. The Programme enables experiential learning in a sensitive and meaningful way and can be useful for training in other institutions.

**Electronic supplementary material:**

The online version of this article (doi:10.1186/s12909-017-0858-7) contains supplementary material, which is available to authorized users.

## Background

Training a medical workforce to meet the needs of diverse societies requires not only the active recruitment of under-represented minorities, but ensuring that all graduates have competencies to work effectively in different communities. The importance of teaching diversity and cultural competency in medical curricula is well recognised [1–7]. Some studies have shown that the teaching of culture and diversity can positively influence change [8–10], while others have not [11–13]. Whilst the patient-centred model is often used as a framework for cultural competency training [14], recent research suggests that the inclusion of the social context and social determinants of health of patients through an intersectional framework would be valuable [15]. The Pacific Immersion Programme is an innovative approach to cultural competency training in a New Zealand medical school which enables the students to learn about the health of people within their own social and community setting.

New Zealand is located in the Southwestern Pacific Ocean, with a historical influence as a colonial power over several of its Pacific Island neighbours [16]. During a time of economic prosperity in New Zealand in the 1950s, labourers were actively recruited from these islands [17, 18]. However, following the economic recession of the 1970s and a reduced demand for unskilled labour, Pacific communities experienced economic hardship. Pacific peoples currently make up 7.4% of the total population of New Zealand. They are a heterogeneous group of different ethnicities who suffer disproportionately from poor health outcomes compared to New Zealand’s population overall [19–23]. Pacific peoples die younger and have higher mortality and morbidity rates for many illnesses compared with the total population. For example, cardiovascular disease is the main cause of death. Mortality from cardiovascular and cerebrovascular diseases is higher in Pacific peoples compared with the total population. There are also socio-economic differences where 27% Pacific peoples live in severe hardship compared to 8% of the total population. They are more likely to be unemployed, have lower educational achievements, lower income, less likely to own their own homes and more likely to under-utilise health care services. It is a young population that is predicted to grow to 10% of the total population by 2021 [19]. Only one percent of all medical doctors in New Zealand identify themselves as having a Pacific ethnicity [24], therefore most Pacific patients are likely to treated by a non-Pacific doctor.

Details about how the Pacific Immersion Programme was developed have been previously reported [25]. In brief, at the Dunedin School of Medicine (University of Otago, New Zealand) students in their fourth year of training are invited to spend a weekend living with a local Pacific family. There are approximately 80 students in fourth year. A number of Pacific ethnic groups host the medical students each year. The students are welcomed during a formal cultural process into the local community. Following this, they are accepted as family and part of the community. The University has a Pacific Advisory Group (PAG) that provides guidance on Pacific developments and engagements. The PAG members consist of Pacific community leaders and University Pacific leaders. Relevant issues related to Pacific developments which include the running of this programme are discussed at their meetings. University staff work closely with community coordinators identified by their respective communities to support the programme. The academic convener of the programme is a leader in the local Pacific community. The establishment of this programme was made possible through the strong existing relationships between University Pacific academic staff and local Pacific communities. Medical students receive prior preparation for this programme in the earlier years of their medical training through a lecture series. The lectures establish foundational knowledge about Pacific peoples and the relevant socio-economic determinants of health. In the fourth year of training, students are informed about the cultural immersion programme at the beginning of the academic year. Prior to the programme, students receive tutorials on the key objectives, Pacific cultural protocols, specific information about the community they will be attached to, and expectations of them in this context. Leaders from the local community meet with the students at the University prior to the programme. This initial engagement provides students with the opportunity to ask questions, seek clarity about any aspects of the programme including expectations from the community. The objectives of the programme are:

- Objective 1: Experience living in a Pacific family environment in New Zealand;
- Objective 2: Learn from the community and appreciate their realities and health context;
- Objective 3: Appreciate how culture, beliefs and socio-economic environment influence health and health outcomes;
- Objective 4: Identify areas where health professionals can support positive outcomes;
- Objective 5: Reflect and re-evaluate personal beliefs, assumptions and/or prejudices.

The students are required to complete a reflective essay following their immersion programme. The completion of the essay is compulsory and all students are expected to complete this, as it is part of their summative assessment. This research evaluates the usefulness of this programme for the cultural competency training of medical students at the University of Otago, Dunedin School of Medicine.

## Methods

Two approaches were used. The reflective essays completed by 235 students from 2011 to 2013 were analysed, and the 2011 cohort was invited to complete a pre and post-programme questionnaire. Approximately 60% percent of the total cohort were female, ages ranged from 20 to 39 years, and 70% between 20 and 25 years of age.

### Reflective essays

Both deductive and inductive analyses were undertaken using the same data set but by different authors. For the deductive analysis, the reflective essays written by 235 students (six of whom identified with a Pacific ethnicity) who participated in the programme from 2011 to 2013 were assessed against the programme’s objectives. Excerpts from students’ reflective essays are presented.

The first 79 essays that were chosen for the analysis were the first 79 (of 235) from the randomly numbered essays that had been previously analysed by another author. No new themes were generated from the 35th essay analysed, indicating saturation was achieved. A random selection of the remaining essays (10%) were analysed with no new themes identified. This subset was not included in the analysed data. The initial coding included all first-hand (student) and second-hand (Pacific community members) statements related to observations from the immersion experience. Statements about Pacific communities that were not derived from the immersion experience, or that resulted from student de-briefs/discussions during/post the immersion experience, were not included. Any statements where it was unclear whether they were related to the immersion experience were excluded. In the initial coding, positive and negative observations/assertions related to the same item were coded separately, but then aggregated as themes were refined, e.g. “social structure and/or culture as a determinant (constructive or obstructive) in allowing (positive or negative) behaviour change”. For the frequency analysis, each coded statement from an essay was counted as one instance. Repeats of the same coded statement in an essay only counted as one instance. Ethnicity frequencies (and association of statements with ethnicities) was based on what was reported in the essay.

### Questionnaire

Students in the 2011 cohort were invited to complete a pre and post-programme questionnaire to describe their views on cultural issues, knowledge of Pacific culture and the main health challenges for Pacific peoples. Questions are reported on a five point Likert scale. These likert scales are presented as a summary percent of positive responses to each question. The *p*-value from mixed model analyses, were performed on the Likert responses to test any difference between pre and post-programme responses. All analyses were preformed using SAS 9.2.

Ethical approval was obtained through the University of Otago Human Ethics Committee. All authors contributed to the analyses, interpretation and writing of this manuscript. FS is the lead author and was responsible for the deductive analysis, contributed to the interpretation of all data and coordinated the development of the manuscript. TZ was responsible for the inductive analysis, interpretation of data and contributed to writing the manuscript. JK was responsible for the statistical analysis of data from the questionnaire, and contributed to the interpretation of data and the development of the manuscript. SC contributed to the literature review, data interpretation and writing the manuscript.

## Results

### Part 1: Deductive analysis

#### Objective 1: Experience living in a Pacific family environment in New Zealand

The students found the programme to be a unique and valuable learning environment which helped them to understand the factors that influence the health of this community. In addition, students reflected on their contribution to improving health outcomes as health professionals in the future.

*“In preventative and social medicine we are taught, frequently, about the social determinants of disease, with the Dalgren and Whitehead diagram repeated ad nauseam. So whenever I see that diagram I tend to switch off without giving it more than a cursory glance. But this weekend I gained a true insight into the real social determinants of health, disease and wellbeing.” (student 15, female)*

*“The patient is your best teacher. Through this weekend I have gained an incredible amount of insight. I am so grateful … otherwise I might have missed out on an immensely valuable opportunity.” (student 188, female)*

*“The immersion weekend was probably one of the most relevant experiences so far as a medical student in term of discovering the inner workings of community and understanding the important role I will play as a doctor to meet their needs.” (student 158, female)*

#### Objective 2: Learn from the community and appreciate their realities and health context

People in the community were good teachers and effective engagement as good practice was valued. The importance of including patients in the management of their health and understanding their context, including respect for their views and perspectives on health, were highlighted.

*“This short period with my host family has helped me understand the Pacific culture better because this provided the opportunity for them to directly speak to me and let me know what they really think I should understand about them.” (student 10, female)*

*“We need to involve them in the management of their own treatment, allow them to feel more in command of their health status … and they need to be offered respect for any cultural and personal beliefs that they might possess.” (student 105, female)*

*“I did not find the weekend easy … what I witnesses and lived with this weekend were beyond cultural diversity, it was serious issues associated with poverty.” (student 17, female)*

#### Objective 3: Appreciate how culture, beliefs and socio-economic environment influence health and health outcomes

Students learnt about the complexities and diversities within Pacific communities; their priorities, beliefs, the influence of the socio-economic environment on people’s choices and how these impact on health and health outcomes.

*“People are under an obligation to financially take care of the community. While this makes it extremely difficult for one individual to amass wealth, it creates an amazing warmth and support within the community. They put spiritual and community health far above their physical or financial well-being.” (student 51, male).*

*“Throughout medical school we have heard about fast food consumption and the obesity epidemic particularly in Pacific people. However it was not until I saw it first-hand that I understood the extent to which this was a problem.” (student 92, female)*

*“One of the things that struck me was the vast diversity within the Pacific Islands population. It was not a one size fits all rule for these communities.” (student 67, male)*

#### Objective 4: Identify areas where health professionals can support positive outcomes

The students identified possible challenges in efforts to improving health outcomes in communities and the role of the physician in society. Different approaches may be required to achieve realistic outcomes. There is a recognition of the need for a diverse health workforce to meet the needs of diverse communities.

*“The programme made me realise just how difficult it would be for Pacific Islanders as they are constantly surrounded by unhealthy food at gatherings, and cannot afford to buy healthy food. I had always thought I would just educate my patients on how to eat healthy, but I now realise that the problem is much more complex.” (student 106, female)*

*“In the future, as a health professional, it is important that I bear in mind all the aspects behind a person: the home, the job, the financial context, the social environment, and the culture with its own strengths and style. By having an increased awareness of the possibility and variability of these I will be more likely to enquire about them and spend the time getting to know the patients context a little better, which should enable me to offer more appropriate and holistic medical care. I am very grateful for the opportunity this programme offered me.” (student 90, female)*

*“The need for a strong Pacific people health workforce seem even more important. We need to actively engage with existing Pacific people health networks if we are to move beyond paying lip service to cultural competence.” (student 97, female).*

#### Objective 5: Reflect and re-evaluate personal beliefs, assumptions and/or prejudices

Students recognised the influence their own beliefs and background can have on the patient-doctor relationship. They learnt that in addition to the various challenges, there are also strengths that culture and communities contribute to peoples’ health and well-being.

*“The most thought provoking part of the week-end was how my prior assumptions and perspectives on Pacific health were challenged. Our Public Health teaching had communicated on negatives such as smoking and alcohol which I did see. However, I saw positive factors too … the strong sense of community and support networks.” (student 22, male)*

*“The recognition that my own culture and the impact it has on my views and health practices is also critical. These issues need to be taken into consideration as we aim to become more culturally competent doctors of the future.” (student 74, female)*

*“One of the great things about being a doctor is the opportunity and necessity for life-long learning. Self-reflection is crucial for a doctor’s learning. Doctors must constantly ask themselves what they learnt from their experiences and how to do things better in the future. The Pacific Immersion Programme was a fantastic opportunity for me to learn and practice new skills to communicate effectively across cultures.” (student 107, male)*

### Part 2: Inductive analysis

Of the 79 essays assessed in the inductive analysis, 27% represented immersion experiences with Tongan families, 23% with Samoan families, 18% Cook Islanders, 11% Fijians, 17% mixed Pacific or other Pacific peoples, and 4% had no clearly identified ethnicity. Language as a factor in health outcomes was a strong theme in the essays, but was prompted in the instructions for the essay and was not included as a theme. The most common emergent themes were the strength of community and social networks (and their influences), the role of food, and issues related to financial constraints on health (Table 1).

**Table 1:** High frequency observations from the immersion experience

No.	Themes	Incidence^a^
1	Strong family and/or social network	68%
2	Church/religion/spirituality as an important aspect of daily activities	55%
3	Co-occurrence of 1 and 2	44%
4	Social structure and/or culture as a determinant (constructive or obstructive) in allowing (positive or negative) behaviour change	60%
5	Food (quality, type, portion size) as a (mainly negative) determinant of health	55%
6	Financial constraints	44%

^a^Frequency of reporting in the reflective essays

Some themes were observed at different frequencies depending on the ethnic context (Table 2). For example, kava, a ceremonial drink, is used only by some ethnic groups.

**Table 2:** Themes observed at different frequencies depending on the ethnic context

No.	Population A (Incidence^a^)	Themes	Population B (Incidence ^a^)
1	77%	Social structure and/or culture as a determinant (permissive or obstructive) in allowing (positive or negative) behaviour change	30%
2	54%	Church/religion/spirituality as an important aspect of daily activities	26%
3	23%	Kava drinking	0%

Note. Populations A and B represent different combinations of two of the four largest Pacific ethnicities. Combinations of populations A and B are the same for Nos. 1 and 2. For No. 3, A and B are a different combination of Pacific ethnic groups

^a^Frequency of reporting in the reflective essays

Table 3 outlines less common themes which were more varied in nature. These include patient-doctor relationships, priorities, use of traditional medicine, and the family context when caring for children.

**Table 3:** Low frequency (<7%) observations from the immersion experience

No.	Themes
1	Power differential between patient and doctor
2	Low priority on health care (related to personal health or in seeking medical attention when required)
3	Desirability of family support during a consultation
4	The use of traditional medicines and/or spiritual effectors (positive or negative) on health
5	Shared care of children, such that adults accompanying a child (seeking health care) may not be biologically related nor have a complete understanding of the history of the child

### Part 3: Survey

The Pacific Immersion Programme increased students’ comfort levels in cross-cultural engagement and their understanding of the importance of a patients’ cultural perspectives in health care provision (Table 4). Few students agreed effective health care was possible without understanding their patient’s cultural perspective. Students’ self-reported knowledge about Pacific cultural values, protocols, traditional beliefs and the main health challenges increased significantly after the programme.

**Table 4:** Students’ responses before and after the Programme as “agree” or “strongly agree” with each statement

Statements	*n*	Pre %	Post %	*p*-value
There are differences in my upbringing compared to Pacific Peoples	77	79.2	74.0	0.2897
I am interested in learning more about Pacific Peoples	78	92.3	91.9	0.9657
Understanding a Pacific patient’s cultural perspective on health is essential to the provision of effective health care	77	89.6	92.1	0.0402^a^
I am conscious of cultural differences when interacting with people from other cultures	78	76.9	90.9	0.0041^a^
I am open to learning new things in a different environment	78	92.3	94.8	0.0458^a^
I enjoy interacting with people from different cultures	78	89.8	97.4	0.0169^a^
I am open to living in a culture that is unfamiliar to me	78	65.4	80.3	0.0175^a^
I show the same regard for other cultural points of view as I do for my own culture	78	91.0	93.5	0.2964
I know about Pacific cultural values and/or protocols	78	21.8	89.5	<0.0001^a^
I know about Pacific traditional health beliefs and/or practices	78	10.3	77.9	<0.0001^a^
I know about the main health challenges of Pacific peoples living in NZ	70	22.9	65.1	<0.0001^a^

^a^Statistically significant

## Discussion

The need to train a competent health workforce to meet the needs of increasingly diverse communities is widely acknowledged [26–28]. Responsiveness to the patient’s socio-cultural context is critical [29]. Researchers have examined cultural competency and how best to train health professionals to meet the needs of diverse communities [8, 14, 28]. It is recognised that provider bias influences health care outcomes [30]. Students are often unaware of their own biases [31] and training to address such biases may not be well received [32]. This immersion programme enables students to experience the realities and context of a minority community “from the inside”. Being part the community gave students the privilege to observe and learn about the challenges minority communities experience in efforts to improve their health. The Pacific Immersion Programme allows student learning within a patient-centred cultural care model [14] in a community setting. The model emphasises the need for doctors to recognise their own biases and assumptions. In addition to this, it incorporates aspects of the intersectional framework approach [15] which requires doctors to appreciate the importance of the patient’s social context (the social determinants of health).

The Pacific Immersion Programme was developed to provide an environment where students can learnt about the key factors that impact on the health of a minority community, not as passive observers, but as engaged ‘extended family members’. Its continuity depends on the value for student learning and ongoing community support. The programme was very well received by Pacific communities as they felt empowered in the process and through engagement [33]. This research explored the usefulness for students participating in the immersion programme as a window into the type of learning that can be achieved through this modality and also how it could be best blended with other modes of teaching.

The overwhelming response from the students was that they felt the programme was a unique and valuable learning opportunity, and that they appreciated learning about health issues directly from community members. The survey results supported the programme’s value for learning and improved students understanding of Pacific values, beliefs and the main challenges to the health of Pacific peoples in New Zealand. The students reflected on the need to understand the patients’ perspective, their context, and involving them in their health management. Most of the health concerns students observed were linked to socio-economic realities such as inadequate income, unemployment, low-wage jobs, poor housing and low education. The health concerns and socio-economic determinants of health for Pacific peoples as observed by the students have been documented [20, 22, 34]. The students also highlighted the strong supportive family and community networks, including the role of the church, noting that individuals (probably due to these strong linkages) prioritised their families, community and church commitments over their own personal health. This tied into the observation that Pacific communities expressed their culture, generosity, and hospitality through the provision of food, and the challenges these present when coupled with an obesogenic diet. Students’ reflections showed an awareness of how their own views and perspectives might influence outcomes. They acknowledged the importance of fairness, inequalities, social responsibilities, environmental influences, reciprocity, cultural understanding and competencies.

The students’ essays are not only a valuable tool for reflective learning from an activity like the Pacific Immersion Programme, they can assist with curriculum development by indicating the range and penetrance of experiences. Themes that are highly penetrant in first person accounts may not require instructor-led, second-hand accounts. Such themes lend themselves better to a moderated peer-to-peer de-brief (which occurs as part of the Pacific Immersion Programme) and synthesis activity. Furthermore, impromptu peer discussions (which were reported in the reflective essays) mean it is likely that these high frequency concepts will permeate through to all members of the class.

However, the level of reporting of observations is not necessarily equal to their frequency or importance in the context of health outcomes. Further, the relevance and prevalence of some sensitive themes in many Pacific groups may be inversely proportional to their reporting frequency in the reflective essays. Thus, important low frequency or unreported themes need to be reinforced through teaching in other settings.

Similarly, there are a subset of themes whereby the level of exposure depends on the specific ethnic group in which the student is immersed. For example, the drinking of kava (root extract with a sedative effect) is more common amongst some Pacific groups. There are some potential negative socio-dynamic issues associated with the practice [35]. Therefore, depending on the placement, a student may think that this is common in all Pacific groups. We note here that the de-identification of ethnicities in Table 2 is deliberate, as the intention is not to ascribe values to a specific Pacific community, but to illustrate the potential effect on learning from the different experiences. Thus, strategies are needed to account for differential learning as a result of specific placements, as students are likely to encounter all Pacific ethnicities in practice.

Results from the analyses of reflective essays and the survey suggest that learning in this context can be an invaluable experience for medical students. Although the programme duration was short, students’ answers to the same questions (before and the after the programme) about the main health challenges, culture, values, beliefs and practices in this community, suggest their knowledge on these areas increased significantly. The process for learning in this context which has a community approach, that is, the “whole village” is involved in the teaching or transfer of knowledge, is likely to be a major contributor to this outcome. Students’ reported also their observations of the strengths within families and communities which can support positive change. Perhaps of greater significance are their reflections of how their perspectives (shaped by their background, cultures, biases, prejudices and assumptions) can influence their approach to the provision of health care. Many students acknowledged the valuable insights they have gained about best practice from this context, was made possible through the generosity of the host communities enabling this learning experience.

## Conclusion

Medical institutions have a responsibility to train a health workforce equipped to meet the needs of the communities they serve. The Pacific Immersion Programme provides a useful learning environment which can enhance the training of doctors to work in diverse communities. It enables students to be engaged learners in this environment from “the inside” rather than an “outsider looking in”. The Programme enables experiential learning in a sensitive and meaningful way and can be useful for other training institutions. The success of such learning programmes depend on the value for student learning, empowering minority communities and ensuring ongoing mutual and trusting relationships between the tertiary institution and these communities.

## Additional files

Additional file 1: Pacific Immersion Programme Questionnaire – (Before). Medical students knowledge and views of Pacific people, health and culture before the Pacific Immersion Programme. (PDF 600 kb)
Additional file 2: Pacific Immersion Programme Questionnaire – (After). Medical students knowledge and views of Pacific people, health and culture after the Pacific Immersion Programme. (PDF 606 kb)
