# Supplementary material for: Training a medical workforce to meet the needs of diverse minority communities
Source: BMC Med Educ. 2017 Jan 21;17:19. doi: 10.1186/s12909-017-0858-7 (PMC5251211; doi:10.1186/s12909-017-0858-7)
Supplement: Additional file 2: — Pacific Immersion Programme Questionnaire – (After). Medical students knowledge and views of Pacific people, health and culture after the Pacific Immersion Programme. (PDF 606 kb) [file 12909_2017_858_MOESM2_ESM.pdf]

Please Print PIP Number in this box

Otago Medical School Pacific Immersion Programme (PIP) Evaluation Questionnaire

Section A:

(Please tick the appropriate corresponding box)

	Strongly Disagree	Disagree	Somewhat Agree	Agree	Strongly Agree
1)There are differences in my upbringing compared to Pacific Peoples	☐	☐	☐	☐	☐
2)I am interested in learning more about Pacific Peoples	☐	☐	☐	☐	☐
3)Physicians can give excellent health care without understanding their patient's cultural background	☐	☐	☐	☐	☐
4)Understanding a Pacific patient's cultural perspective on health is essential to the provision of effective health care	☐	☐	☐	☐	☐
5)I am conscious of cultural differences when interacting with people from other cultures	☐	☐	☐	☐	☐
6)I am open to learning new things in a different environment	☐	☐	☐	☐	☐
7)I enjoy interacting with people from different cultures	☐	☐	☐	☐	☐
8)I am open to living in a culture that is unfamiliar to me	☐	☐	☐	☐	☐
9)I show the same regard for other cultural points of view as I do for my own culture	☐	☐	☐	☐	☐

Section B:

(Please tick the appropriate corresponding box)

	Strongly Disagree	Disagree	Somewhat Agree	Agree	Strongly Agree
1)I know some cultural values and/or protocols of Pacific cultures	☐	☐	☐	☐	☐
2)I know some traditional health beliefs and/or practices in Pacific cultures	☐	☐	☐	☐	☐
3)I know about the main health challenges faced by Pacific Peoples in New Zealand<br>(If you know, please name three you consider to be most important)	☐	☐	☐	☐	☐

I. ____

II. ____

III. ____

(Section B continues over the page)

(Section B continued)

(Please tick the appropriate corresponding box)

	Strongly Disagree	Disagree	Somewhat Agree	Agree	Strongly Agree
4)When addressing Public Health issues in a Pacific community it is important to work with:					
• Individuals	☐	☐	☐	☐	☐
• Families	☐	☐	☐	☐	☐
• Community	☐	☐	☐	☐	☐
5)These are factors that influence access to health services in New Zealand for Pacific Peoples					
• Cost	☐	☐	☐	☐	☐
• Transport	☐	☐	☐	☐	☐
• Language	☐	☐	☐	☐	☐
• Traditional Medicine	☐	☐	☐	☐	☐
• Unemployment	☐	☐	☐	☐	☐
• Level of Education	☐	☐	☐	☐	☐

Please list any other factors that you know influence access to health services in New Zealand for Pacific Peoples

________________________________ ________________________________
________________________________ ________________________________
________________________________ ________________________________
________________________________ ________________________________
________________________________ ________________________________

Section C:

(Please tick the appropriate corresponding box)

	Strongly Disagree	Disagree	Somewhat Agree	Agree	Strongly Agree
1) I feel confident using Pacific cultural greetings when speaking with Pacific Peoples	☐	☐	☐	☐	☐

Please list the communication skills you have that will help you to work with Pacific patients, families and communities

________________________________ ________________________________
________________________________ ________________________________
________________________________ ________________________________
________________________________ ________________________________
________________________________ ________________________________

(Please turn page over for Section D)

Section D:

1) Did this experience give you confidence to work with Pacific communities?

____
____
____
____
____
____
____
____

2) What were the best aspects of this experience for you?

____
____
____
____
____
____
____
____

3) What changes would you suggest to enhance your learning?

____
____
____
____
____
____
____
____

4) Any other comments

____
____
____
____
____
____
____
____

Thank you for taking the time to complete this survey.
We appreciate your involvement in the programme and your feedback.
